# Verification of a Stiffness-Variable Control System with Feed-Forward Predictive Earthquake Energy Analysis

**DOI:** 10.3390/s21227764

**Published:** 2021-11-22

**Authors:** Tzu-Kang Lin, Tappiti Chandrasekhara, Zheng-Jia Liu, Ko-Yi Chen

**Affiliations:** Department of Civil Engineering, National Yang Ming Chiao Tung University, Hsinchu 30010, Taiwan; tchandrasekhar92@gmail.com (T.C.); zyp6035@yahoo.com.tw (Z.-J.L.); ericchen870529@g.ncu.edu.tw (K.-Y.C.)

**Keywords:** semi-active control, near-fault earthquake, ground motion characteristics, potential energy

## Abstract

Semi-active isolation systems with controllable stiffness have been widely developed in the field of seismic mitigation. Most systems with controllable stiffness perform more robustly and effectively for far-field earthquakes than for near-fault earthquakes. Consequently, a comprehensive system that provides comparable reductions in seismic responses to both near-fault and far-field excitations is required. In this regard, a new algorithm called Feed-Forward Predictive Earthquake Energy Analysis (FPEEA) is proposed to identify the ground motion characteristics of and reduce the structural responses to earthquakes. The energy distribution of the seismic velocity spectrum is considered, and the balance between the kinetic energy and potential energy is optimized to reduce the seismic energy. To demonstrate the performance of the FPEEA algorithm, a two-degree-of-freedom structure was used as the benchmark in the numerical simulation. The peak structural responses under two near-fault and far-field earthquakes of different earthquake intensities were simulated. The isolation layer displacement was suppressed most by the FPEEA, which outperformed the other three control methods. Moreover, superior control on superstructure acceleration was also supported by the FPEEA. Experimental verification was then conducted with shaking table test, and the satisfactory performance of the FPEEA on both isolation layer displacement and superstructure acceleration was demonstrated again. In summary, the proposed FPEEA has potential for practical application to unexpected near-fault and far-field earthquakes.

## 1. Introduction

Continual improvements in earthquake engineering have allowed many structures to meet seismic design requirements for reducing earthquake-induced damage or collapse that exceeds the allowable range of construction. In conventional seismic design, structural strength is utilized to dissipate energy, which causes a considerable amount of plastic deformation in the frame. Extensive maintainability and damage reduction are essential under excessive seismic forces. Therefore, robust control systems are crucial for reducing seismic energy and structural responses in terms of displacement and acceleration [1].

Kobori et al. [2] were the first to investigate earthquake isolation systems according to the concept of variable stiffness, theoretically and experimentally demonstrating its feasibility [3]. In the case of variable stiffness controllable isolation systems, optimum variable stiffness will make the structure in controllable under the earthquake excitations without further increase of acceleration. Narasimhan et al. [4] proposed a semi-active variable-stiffness control system mainly composed of four springs arranged in a diamond shape and an actuator requiring minimal electrical power for changing the spring angle. Alternatively, Nagarajaiah and Sahasrabudhe [5] proposed a semi-active isolation system called semi-active independently variable stiffness (SAIVS), which has four springs arranged in a horizontal rhombus configuration and by adjusting the angle between the springs of the SAIVS, the efficacious stiffness of the isolation system can be achieved to suppress the base displacements. Similarly, Sahasrabudhe and Nagarajaiah [6] conducted an experimental study on a small scale SAIVS device and demonstrated that, by switching the stiffness continuously, a non-resonant state can be achieved with both the displacement and acceleration responses being reduced.

**Figure 1:**
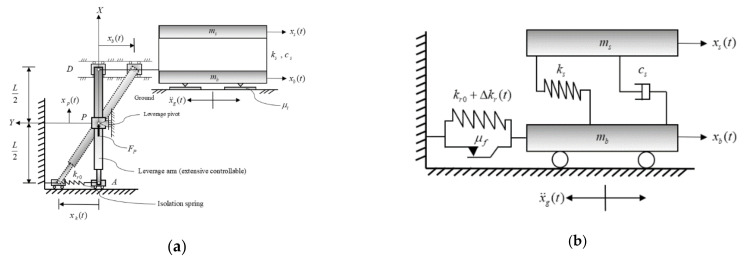

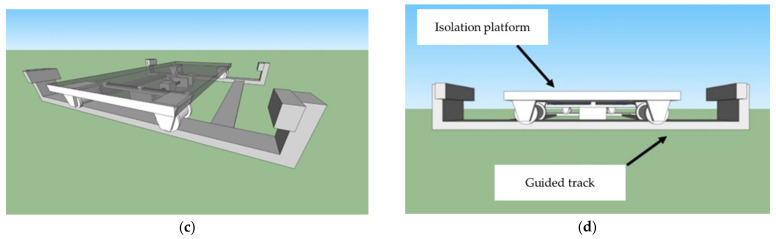
Schematic diagram of LSCIS isolation system. (**a**) Physical model of the LSCIS isolation system. (**b**) Mathematical model of the LSCIS isolation system. (**c**) Drawing of the LSCIS in 3D. (**d**) Side view of the LSCIS mechanism.

Yang et al. suggested that the semi-active stiffness damper constitutes a component of variable stiffness [7]. Zhou and Liu developed a tunable high-static–low-dynamic-stiffness system for variable passive control or semi-active control with a mechanical spring connection [8,9]. Lu et al. developed a semi-active isolation system called the leverage-type stiffness controllable isolation system (LSCIS) [10]. In a subsequent study, the researchers found the least input energy method (LIEM) to be successful in controlling the stiffness of the LSCIS [11]. However, the performance of LIEM was deteriorated under pulse-like ground motion record. To overcome this issue, Newmark [12] used the vertical-to-horizontal ratios of peak ground acceleration (PGA) to detect near-fault earthquakes. Subsequently, techniques based on wavelet analysis were developed [13,14,15] to identify the characteristics of near-fault earthquakes, classify tremors as pulse-like or non-pulse-like earthquakes, and analyze structural responses to near-fault earthquakes. Mavroeidis et al. explored the effects of near-fault earthquakes on elastic and inelastic single-degree-of-freedom structures [16]. Chen et al. developed the minimum energy weighting (MEW) method to control the displacement of the isolation layer [17]. However, as only two control modes were provided by the MEW method, a rapid prediction system to identify the earthquake ground motion characteristics as well as to suppress the structural vibrations under both the near-fault and far-field earthquake excitations is required. The main aim of this study is to mitigate the structure response by controlling the semi-active isolation system under the near-fault and far-field earthquakes, and the optimal stiffness is achieved by adjusting the pivot point of the LSCIS (point P in Figure 1). As the existing control systems are mostly effective under far-field earthquakes only, a rapid prediction system called FPEEA is constructed in this study to identify the earthquake ground motion characteristic as well as to suppress the structural vibrations under both the near-fault and far-field earthquake excitations.

The remainder of this paper is organized as follows. In Section 2, a comprehensive derivation for the governing equation of motion of a structure with the LSCIS is presented. In Section 3, the seismic velocity energy index is defined, and the Feed-Forward Predictive Earthquake Energy Analysis (FPEEA) algorithm is proposed. In Section 4, the model parameters, parameter optimization process, and numerical simulations developed to benchmark the proposed system are presented. In Section 5, details of the experimental setup and the verification results are presented. Finally, the conclusion is drawn in Section 6.

## 2. Analytical Model for Structures with the LSCIS

### 2.1. Equation of Motion for the LSCIS

A schematic of the LSCIS is shown in Figure 1. The controllable stiffness $k_{r}\left(t\right)$ is presented in terms of two components: the uncontrollable support stiffness $k_{r0}$ and the controllable time-variant stiffness $\Delta k_{r}\left(t\right)$ [18].

(1) $$k_{r}\left(t\right)=k_{r0}+\Delta k_{r}\left(t\right)$$

The dynamic equation of motion for the LSCIS is derived from Lagrange’s equation and converted into a state-space equation by employing a step-by-step integration method as follows [19]:

(2) $$z\left[k+1\right]=A_{d}z\left[k\right]+B_{d}D_{d}z\left[k\right]\Delta k_{r}\left[k\right]+E_{d}{\ddot{x}}_{g}\left[k\right]$$

(3) $$A_{d}=e^{A\Delta t}\;B_{d}=A^{-1}\left(A_{d}-I\right)\;B\;E_{d}=A^{-1}\left(A_{d}-I\right)\;E$$

(4) $$A=\left[\begin{array}{cc} -M^{-1}C & -M^{-1}K\\ I & 0 \end{array}\right]z\left(t\right)=\left\{\begin{array}{c} {\dot{x}}_{s}\left(t\right)\\ {\dot{x}}_{b}\left(t\right)\\ x_{s}\left(t\right)\\ x_{b}\left(t\right) \end{array}\right\}B=\left\{\begin{array}{c} 0\\ -1/m_{b}\\ 0\\ 0 \end{array}\right\}E=\left\{\begin{array}{c} -1\\ -1\\ 0\\ 0 \end{array}\right\}$$

where $z\left[k+1\right]$ is the state vector at step $\left[k+1\right]$ and $A_{d}$, $B_{d}$, $D_{d},$ and $E_{d}$ are discrete-time forms of the system matrix ***A***, the support matrix ***B***, the isolation matrix ***D*,** and the excitation matrix ***E***, respectively. In Equation (4), ***M***, ***C***, and ***K*** are the masses, damping, and stiffness matrices of the isolated structure, respectively. These parameters can be expressed as follows:

(5) $$M=\left[\begin{array}{cc} m_{s} & 0\\ 0 & m_{b} \end{array}\right]K=\left[\begin{array}{cc} k_{s} & -k_{s}\\ -k_{s} & k_{s}+k_{r0} \end{array}\right]C=\left[\begin{array}{cc} c_{s} & -c_{s}\\ -c_{s} & c_{s}+c_{r} \end{array}\right]D_{d}=\left[\begin{array}{cccc} 0 & 0 & 0 & 1 \end{array}\right]$$

where $m_{s}$ and $m_{b}$ represent the masses of the superstructure and isolation layer, respectively; $k_{s}$ is the stiffness of the superstructure; $c_{s}$ and $c_{r}$ are the damping coefficients of the superstructure and isolation layer, respectively; ${\ddot{x}}_{g}\left(t\right)$ is the ground acceleration; and $x_{s}\left(t\right)$ and $x_{b}\left(t\right)$ represent the relative displacements of the superstructure and isolation layer, respectively.

As presented in Equation (2), $z\left[k+1\right]$ can be determined from the state vector $z\left[k\right]$, the ground acceleration ${\ddot{x}}_{g}\left[k\right]$, and the incremental stiffness $\Delta k_{r}\left[k\right]$ whereas ${\ddot{x}}_{g}\left[k\right]$ and $z\left[k\right]$, which can be determined according to the response in the previous step $\left[k-1\right]$, are known values in the *k*th step. On the basis of the LSCIS (Figure 1a), the relationship between the incremental stiffness of the isolation layer $\Delta k_{r}\left[k\right]$ and the pivot displacement $x_{p}\left[k\right]$ shown in Equation (8) can be derived from Equations (6) and (7) [18]:

(6) $$x_{k}\left[k\right]=\frac{0.5L+x_{p}\left[k\right]}{0.5L-x_{p}\left[k\right]}x_{b}\left[k\right]\;\;\;$$

(7) $${\left(\frac{0.5L+x_{p}\left[k\right]}{0.5L-x_{p}\left[k\right]}\right)}^{2}k_{r0\;}x_{b}\left[k\right]=\left(1+\frac{2Lx_{p}\left[k\right]}{{\left(0.5L-x_{p}\left[k\right]\right)}^{2}}\right)k_{r0\;}x_{b}\left[k\right]$$

(8) $$\Delta k_{r}\left[k\right]=\left[\frac{2Lx_{p}\left[k\right]}{{\left(0.5L-x_{p}\left[k\right]\right)}^{2}}\right]k_{r0}$$

where *L* is the length of the leverage.

As presented, the incremental stiffness of the isolation layer, $\Delta k_{r}\left[k\right]$, can be controlled by changing the pivot displacement $x_{p}\left[k\right]$, which ranges from 0.0505L to −0.191 L. The above-mentioned procedure can be figured out in the Figure 1c where the position of the pivot point is adjusted along the black leverage arm.

### 2.2. MEW Method

The derivation of the optimal MEW method [17] is mainly based on the LIEM [19]. The optimal weighting values of the kinetic and potential energies of the superstructure and isolation layer are calculated, and the controllable stiffness is adjusted to reduce the structural response to seismic excitation. To optimize the stiffness under the MEW, an energy index $J\left[k+1\right]$ is defined as follows:

(9) $$J\left[k+1\right]=E_{k}\left[k+1\right]+\frac{R}{2}\Delta k_{r}{\left[k\right]}^{2}+QE_{p}\times E_{p,sup}\left[k+1\right]+E_{p,iso}\left[k+1\right]$$

where $E_{k}\left[k+1\right]$ is the kinetic energy of the structure for the (*k* + 1)th step, *R* is the pivot limit parameter, $\Delta k_{r}\left[k\right]$ is the incremental stiffness of the isolation layer, $QE_{p}$ is the weighting of the potential energy, $E_{p,sup}\left[k+1\right]$ is the energy of the superstructure in the (*k* + 1)th step, and $E_{p,iso}\left[k+1\right]$ is the isolation layer energy in the (*k* + 1)th step. The optimal increment in the isolation layer stiffness $\Delta k_{r,opt}\left[k\right]$ must minimize $J\left[k+1\right]$ to satisfy Equation (9).

(10) $$\frac{d\left(J\left[k+1\right]\right)}{d\left(\Delta k_{r}\left[k\right]\right)}=0$$

Through detailed calculations, Equation (10) can be rewritten as follows:

(11) $$\left(a_{1}+R+QE_{p}\times b_{2,sup}+QE_{p}\times b_{2,iso}\right)\Delta k_{r}+\left(a_{2}+QE_{p}\times b_{3,sup}+QE_{p}\times b_{3,iso}\right)=0$$

Furthermore, Equation (11) is rearranged to obtain $\Delta k_{r,opt}\left[k\right]$ as follows:

(12) $$\Delta k_{r,opt}\left[k\right]=\frac{-\left(a_{2}+QE_{p}\times b_{3,sup}+QE_{p}\times b_{3,iso}\right)}{a_{1}+R+QE_{p}\times b_{2,sup}+QE_{p}\times b_{2,iso}}$$

The coefficients in Equations (11) and (12) are defined as follows:

(13) $$a_{1}\left[k\right]=z{\left[k\right]}^{T}B_{1d}{}^{T}D_{2}{}^{T}MD_{2}B_{1d}z\left[k\right]\;a_{2}\left[k\right]=z{\left[k\right]}^{T}B_{1d}{}^{T}D_{2}{}^{T}MD_{2}\left(A_{d}z\left[k\right]+E_{d}{\ddot{x}}_{g}\left[k\right]\right)+DMD_{2}B_{1d}z\left[k\right]{\dot{x}}_{g}\left[k\right]\;b_{2,iso}\left[k\right]=B_{1dL}z\left[k\right]\left(A_{dL}z\left[k\right]+E_{dL}{\ddot{x}}_{g}\left[k\right]\right)K_{r0}\;b_{3,iso}\left[k\right]=0.5{\left(A_{dL}z\left[k\right]\right)}^{2}k_{r0}+0.5\left(E_{dL}{\ddot{x}}_{g}\left[k\right]\right)k_{r0}+A_{dL}z\left[k\right]E_{dL}{\ddot{x}}_{g}\left[k\right]k_{r0}\;b_{2,sup}\left[k\right]=B_{1dL}z\left[k\right]\Delta k_{r}(2\left(A_{dL}z\left[k\right]+E_{dL}{\ddot{x}}_{g}\left[k\right]\right)+\left(A_{dT}z\left[k\right]+E_{dT}z\left[k\right]\right))\;+B_{1dT}z\left[k\right]\;\Delta k(2\left(A_{dT}z\left[k\right]+E_{dT}{\ddot{x}}_{g}\left[k\right]\right)+\left(A_{dL}z\left[k\right]+E_{dL}z\left[k\right]\right))\;b_{3,sup}\left[k\right]=0.5{\left(A_{dL}z\left[k\right]\right)}^{2}k_{s}+0.5\left(E_{dL}{\ddot{x}}_{g}\left[k\right]\right)k_{s}+A_{dL}z\left[k\right]E_{dL}{\ddot{x}}_{g}\left[k\right]k_{s}\;+0.5{\left(A_{dT}z\left[k\right]\right)}^{2}k_{s}+0.5\left(E_{dT}{\ddot{x}}_{g}\left[k\right]\right)k_{s}+A_{dT}z\left[k\right]E_{dT}{\ddot{x}}_{g}\left[k\right]k_{s}\;-0.5A_{dT}z\left[k\right]A_{dL}z\left[k\right]k_{s}-0.5E_{dT}{\ddot{x}}_{g}\left[k\right]E_{dL}{\ddot{x}}_{g}\left[k\right]k_{s}\;-0.5A_{dT}z\left[k\right]E_{dL}{\ddot{x}}_{g}\left[k\right]k_{s}-0.5A_{dL}z\left[k\right]E_{dT}{\ddot{x}}_{g}\left[k\right]k_{s}\;A_{dT}=D_{T}A_{d},\;B_{1dT}=D_{T}B_{1d},\;B_{1dL}=D_{L}B_{1d},\;E_{dT}=D_{T}E_{d},\;E_{dL}=D_{L}E_{d}$$

## 3. The FPEEA Algorithm

Structural displacement amplification is a phenomenon observable in the force of near-fault earthquakes; thus, the MEW is developed by further considering the potential energy [19]. However, because only two control modes are adopted, the method may not be robust under individual earthquakes. It fails to assign suitable potential energy weightings according to earthquake type. A parameter called the earthquake velocity energy index is defined and employed in the FPEEA to solve this problem.

### 3.1. Earthquake Velocity Energy Index

The identification of near-fault or far-field earthquakes in earthquake engineering is a challenge because the spectral criteria characterizing near-fault and far-field earthquakes are affected by the station location, epicenter distance, and soil conditions. Near-fault earthquakes inherently (1) possess high peak velocity and displacement, (2) have energy concentrated in one or relatively few pulse waves, and (3) exhibit unusually shaped spectra [20,21]. Compared with regular earthquakes, near-fault earthquakes have a narrower frequency and higher peak Fourier amplitude. Considering these characteristics of near-fault earthquakes, the energy distribution of the spectrum can be defined by using Parseval’s formula as follows [20].

(14) $$\int_{-\infty }^{\infty }{\left[{\ddot{x}}_{g}\left(t\right)\right]}^{2}dt=\left(1/2\pi \right)\int_{-\infty }^{\infty }{\left|y\left(\omega \right)\right|}^{2}d\omega$$

where ${\ddot{x}}_{g}\left(t\right)$ represents the ground acceleration and $y\left(\omega \right)$ is the Fourier transform of ${\ddot{x}}_{g}\left(t\right)$.

To calculate the seismic velocity energy, the acceleration term ${\ddot{x}}_{g}\left(t\right)$ in Equation (14) is integrated to a velocity term ${\dot{x}}_{g}\left(t\right)$. The energy distribution of velocity across specific frequency components can be calculated as follows:

(15) $$E_{\Omega }={\left[\int_{-\infty }^{\Omega }\left|y{\left(\omega \right)}^{2}\right|d\omega \right]}^{1/2}={\left[\int_{-\infty }^{\Omega }y\left(\omega \right)y^{*}\left(-\omega \right)d\omega \right]}^{1/2}$$

Studies have indicated that cumulative energy at frequencies between 0 and 5.0 Hz should exceed 97% of the total energy of a near-fault earthquake [21]. Frequencies between 0 and 1.0 Hz contain most of the energy, and the release of energy continues between 1 and 3 Hz. This information can potentially be used to differentiate between near-fault and far-field earthquakes. The cumulative energy at frequencies between 0 and 5.0 Hz should be less than 97% of the total energy of a far-field earthquake. Table 1 and Table 2 list the energy distributions across frequencies for various near-fault and far-field earthquakes, respectively. The 32 typical near-fault and far-field ground motions were selected from an open-source database. The aforementioned tables indicate that the 97% threshold criterion can be used for earthquake classification. For the rapid identification of earthquake ground motion characteristics, the velocity in the first 3 s duration of the earthquake and the corresponding seismic energy are considered.

### 3.2. The FPEEA Control Law

Because the 97% energy threshold criterion can only be used to roughly categorize the earthquake type, an FPEEA control strategy is proposed in this paper. The accumulated energy at frequencies of 0 to 5 Hz is divided into six segments to classify near-fault and far-field earthquakes at different energy ratios. These six segments of near-fault and far-field earthquake excitations with respect to the energy ratios were listed in Table 3 with the collected earthquake numbers of different energy ratios. When the energy ratio is higher than 99%, the likelihood that the tremor is a near-fault and far-field earthquake is 80% and 20%, respectively. The control leverages the probability of occurrence. Subsequently, it proportionally allocates potential energy weighting to near-fault and far-field earthquakes.

The probabilities of potential energy weighting corresponding to velocity–energy ratios are presented in Table 4, where S_1_, C_1_, and C_2_ represent the energy ratio, weighting for near-fault earthquakes, and weighting for far-field earthquakes, respectively. It indicates the probability of near-fault and far-field energy weightings for a particular velocity–energy ratio. To extend the consideration of potential energy to any near-fault or far-field earthquake, different C_1_ and C_2_ values are used in the FPEEA control. The potential energy weighting is rewritten as follows:

(16) $$QE_{p}=C_{1}\cdot QE_{pn}+C_{2}\cdot QE_{pf}$$

where $QE_{pn}$ represents the potential energy weight of a near-fault earthquake and $QE_{pf}$ denotes the potential energy weight of a far-field earthquake. By substituting Equation (16) into Equation (12), the optimal stiffness $\Delta k_{r,opt}\left[k\right]$ can be expressed as follows:

(17) $$\Delta k_{r,opt}\left[k\right]=\frac{-\left(a_{2}\left[k\right]+\left(C_{1}\cdot QE_{pn}+C_{2}\cdot QE_{pf}\right)\right)\times b_{3,sup}+\left(C_{1}\cdot QE_{pn}+C_{2}\cdot QE_{pf}\right)\times b_{3,iso})}{\left(a_{1}\left[k\right]+R+\left(C_{1}\cdot QE_{pn}+C_{2}\cdot QE_{pf}\right)\times b_{2.sup}+\left(C_{1}\cdot QE_{pn}+C_{2}\cdot QE_{pf}\right)\times b_{2,iso}\right)}$$

## 4. Numerical Simulations

Numerical simulations were performed to compare the performance of the proposed FPEEA control law with those of the existing control of a benchmark two-degrees-of-freedom structure. The displacement of the isolation layer and the acceleration of the superstructure were the two primary indices considered.

### 4.1. Time History Inputs and Model Parameters

**Figure 2:**
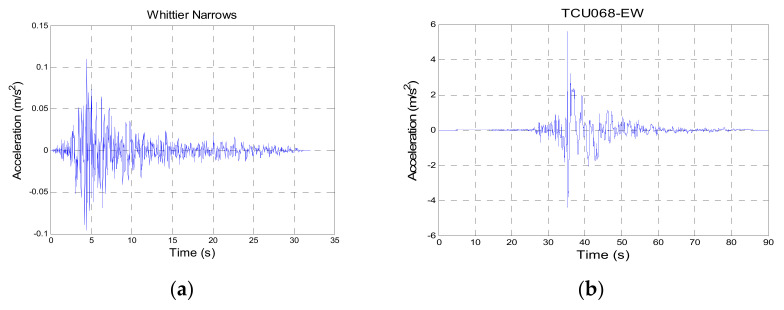
Input time–history data for theoretical simulation. (**a**) Whittier Narrows-01. (**b**) Chi-Chi TCU068-EW.

Two earthquakes were used in the numerical simulations. The considered earthquakes comprised one far-field earthquakes, namely the 1987 Whittier Narrows earthquake (hereafter Whittier Narrows-01), and a near-fault earthquake, namely the 1999 Chi-Chi earthquake (hereafter Chi-Chi TCU068-EW) as shown in Figure 2. The time histories were used to determine the required parameter values related to the control law for earthquake design, and then employed to evaluate the effectiveness of the proposed control law in reducing the structural response. Details of the aforementioned four earthquakes are presented as follows:

(i) Whittier Narrows-01, Glendora-N Oakbank, 1 October 1987, station: A-OAK170, peak acceleration: 0.1099 m/s^2^.
(ii) Chi-Chi, Taiwan, 21 September 1999, station: TCU068-EW, peak acceleration: 5.58 m/s^2^.

The simulated structure was divided into a superstructure and an isolation layer with a mass of 18.66 and 38.445 kg, respectively. The parameters of the superstructure and isolation layer are listed in Table 5.

### 4.2. Parameter Optimization

The parameters R and $QE_{p}$ in $\Delta k_{r,opt}$ were optimized. As indicated in a previous study [18], the potential energy weighting was considered for both near-fault and far-field earthquake excitations but not for individual earthquakes. To extend the potential energy weighting beyond two control modes, different C_1_ and C_2_ values were considered. To optimize the parameters R and $QE_{p}$, the time histories of Whittier Narrows-01 and Chi-Chi TCU068-EW were used as seismic inputs to evaluate the structural response in the form of displacement, velocity, and acceleration. The parameter R ranged from $10^{-12}$ to $10^{-1}$, and $QE_{p}$ ranged from 0 to 300.

**Figure 3:**
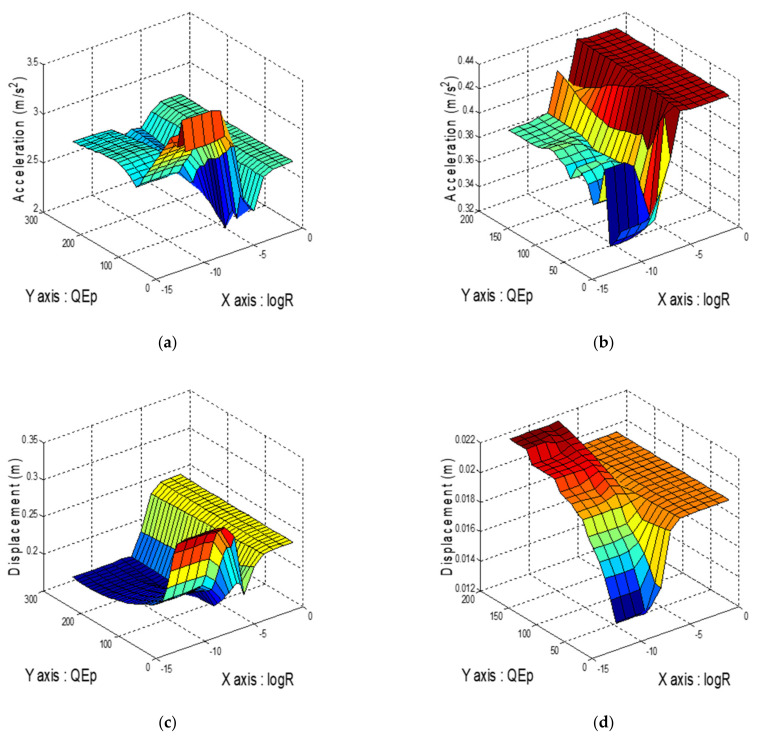
Comparison of different control parameters. (**a**) Superstructure acceleration (Whittier Narrows-01). (**b**) Superstructure acceleration (TCU068-EW). (**c**) Isolation layer displacement (Whittier Narrows-01). (**d**) Isolation layer displacement (TCU068-EW).

Figure 3 displays the numerical simulations of the structural response to Whittier Narrows-01 and Chi-Chi TCU068-EW (PGA = 0.2 g). As shown in Figure 3a, the acceleration response to Whittier Narrows-01 (a far-field earthquake) was the smallest when $R=10^{-6}$ and $QE_{p}=50$. Figure 3c indicates that the minimum displacement was achieved when $R=10^{-8}$ and $QE_{p}=250$. As indicated in Figure 3b, the acceleration response of the superstructure to Chi-Chi TCU068-EW (a near-fault earthquake) increased substantially with an increase in R and $QE_{p}$. For this earthquake, the minimum acceleration response was observed when $QE_{p}=5$ and $R=10^{-9}$. Similarly, as depicted in Figure 3d, the minimum relative displacement was achieved by setting R and $QE_{p}$ as $10^{-9}$ and 5, respectively. According to the results, R was set as $10^{-8}$, and $QE_{pn}$ was determined according to six sets of energy weighting. At the energy weighting value of 300, the control effect did not change significantly. The potential energy weightings for the near-fault earthquakes ($QE_{pn}$) and far-field earthquakes ($QE_{pf}$) were set as 250 and 5, respectively. Through the optimization process shown in Figure 3, the control parameters of the FPEEA can be determined.

### 4.3. Earthquake Simulation Results

The numerical simulation results obtained with the following control approaches were compared: the passive, LIEM, MEW, and FPEEA methods, and the parameters for each control law are listed in Table 6. The generic MEW control was adopted [19]. Passive control refers to the fixation of the lever point at the midpoint with zero-stiffness increments. In the case of a passive state, the isolation period of the LSCIS is approximately 2.12 s as determined using the following equation: $T_{b}=2\pi \sqrt{\left(m_{s}+m_{b}\right)/k_{r0}}$ where $k_{r0}$ represents the stiffness of the isolation layer. Similarly, in the LIEM control ($QE_{p}=0$, $R=10^{-8}$), the kinetic energy of the superstructure and isolation layer is minimized by adjusting the position of the pivot. The difference between the control parameters of the LIEM and MEW methods is that the kinetic energy is minimized when determining the control parameters of the LIEM, whereas both the kinetic energy and potential energy are considered when determining the control parameters of the MEW method.

**Figure 4:**
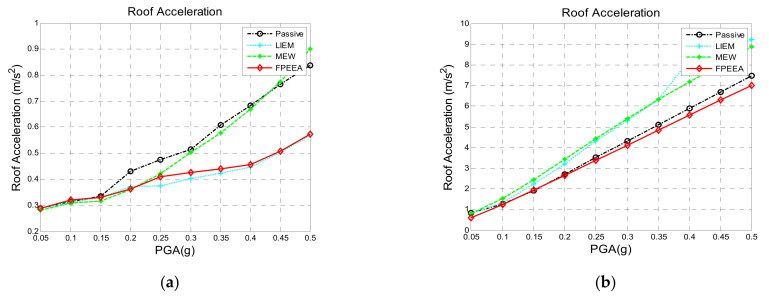

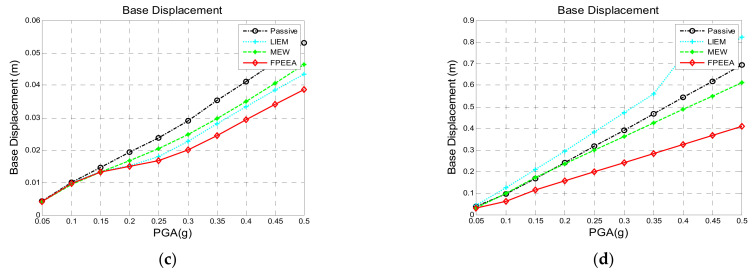
Comparison of the maximum responses of various control laws at different PGA values. (**a**) Superstructure acceleration (WhittierNarrows-01). (**b**) Superstructure acceleration (TCU068- EW). (**c**) Isolation layer displacement (Whittier Narrows-01). (**d**) Isolation layer displacement (TCU068-EW).

Figure 4 displays the results of the numerical simulations for Whittier Narrows-01 (a far-field earthquake) and TCU068-EW (a near-fault earthquake) for different earthquake intensities. As displayed in Figure 4a, similar numerical results were achieved for the far-field earthquake with the FPEEA and LIEM controls. When the earthquake intensity was less than 0.25 g, the FPEEA did not manifest a response. By contrast, when the earthquake intensity exceeded 0.3 g, the superstructure acceleration response was effectively reduced by the FPEEA control, and the effects of the FPEEA control increased with the earthquake intensity. The displacement of the isolation layer under Whittier Narrows-01 at different intensities is illustrated in Figure 4c. As the earthquake intensity increased, the displacement response of the isolation layer significantly reduced by the FPEEA control.

Individual structural responses to Whittier Narrows-01 (PGA = 0.2 g) are listed in Table 7. The peak accelerations of the superstructure under passive control, the LIEM, the MEW method, and FPEEA control were 0.43 (100%), 0.372 (86.6%), 0.36 (83.6%), and 0.362 (84.3%) m/s^2^, respectively. The peak displacement responses of the isolation layer under passive control, the LIEM, the MEW method, and FPEEA control were 0.019 (100%), 0.015 (77.9%), 0.017 (86.4%), and 0.015 (77.3%) m, respectively. The results indicate that the FPEEA control performed favorably in terms of structural response under the excitations of Whittier Narrows-01 (a far-field earthquake). This method robustly reduced the superstructure acceleration and isolation layer displacement responses. As the earthquake intensity increases, larger reductions are expected.

Figure 4b displays the numerical simulations of superstructure acceleration under TCU068-EW (a near-fault earthquake) under changing intensities. The FPEEA control had a higher contribution to superstructure acceleration reduction than did the LIEM and MEW methods when the earthquake intensity was high. Figure 4d, which illustrates the isolation layer displacement response to Chi-Chi TCU068-EW (a near-fault earthquake), indicates that the FPEEA control and MEW method outperformed the LIEM. The FPEEA control is preferable to the MEW method because it definitively reduces the displacement response of the isolation layer.

Details of the responses of the superstructure and isolation layer to Chi-Chi TCU068-EW (PGA = 0.2 g) are shown in Table 8. The peak acceleration responses of the superstructure were 2.710 m/s^2^ (100%), 3.213 m/s^2^ (118.5%), 3.448 m/s^2^ (127.2%), and 2.657 m/s^2^ (98%) under the passive, LIEM, MEW, and FPEEA controls, respectively. Similarly, the isolation layer displacements were 0.242 m (100%) under passive control, 0.294 m (121.8%) under the LIEM, 0.236 m (97.6%) under the MEW method, and 0.156 m (64.8%) under the FPEEA control. Overall, the superstructure acceleration response to Chi-Chi TCU068-EW was effectively controlled by the FPEEA method without amplification. Furthermore, the displacement of the isolation layer was controlled better under the FPEEA control than under the LIEM or MEW method.

## 5. Experimental Verification with Shake Table Tests

**Figure 5:**
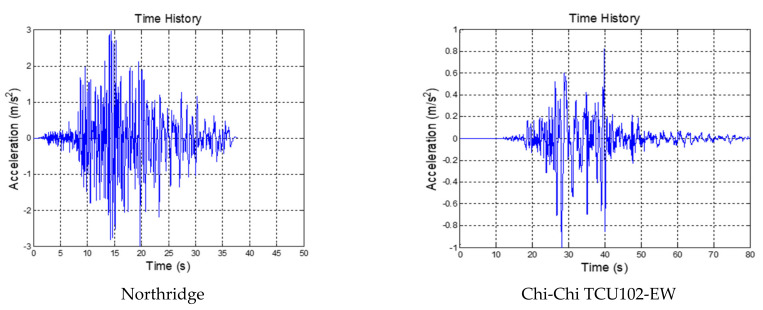
Time history inputs for experimental testing.

To verify the performance of the FPEEA control, a shake table test was conducted for two types of earthquakes, namely the far-field 1994 Northridge earthquake (hereafter Northridge; PGA = 0.20 and 0.30 g) and the near-fault 1999 Chi-Chi earthquake as measured at station TCU102 (hereafter Chi-Chi TCU102-EW; PGA = 0.08 and 0.10 g). The time history inputs of these earthquakes are depicted in Figure 5, and their details are presented as follows:

(i) Northridge-01, 1994, station: Huntington Bch-Waikiki; Mw = 6.69.
(ii) Chi-Chi, Taiwan, 1999/09/21, station: TCU102; Mw = 7.6.

**Figure 6:**
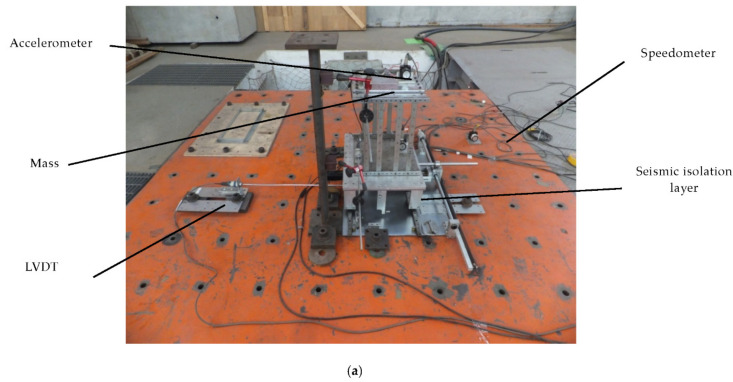

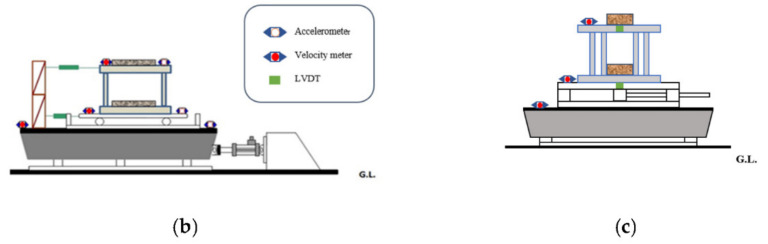
Experimental setup and the instrumentation configuration. (**a**) Assembling of the isolation layer and superstructure. (**b**) Side view of the instrumentation. (**c**) Front view of the instrumentation.

The parameters of the superstructure and isolation layer are listed in Table 9. Figure 6 displays the experimental setup and the Instrumentation configuration. While performing the experimental verification, sensors were deployed to record the responses of the superstructure and the isolation layer. The measurement range of the selected accelerometer, velocity meter, and linear variable displacement transducers (LVDTs) are ±4 g, ±100 kine (cm/sec), and ±300 mm, respectively. With the support of the instrumentation, the practical effectiveness of proposed FPEEA can be verified.

### 5.1. Comparison of the Experimental and Simulation Results

**Figure 7:**
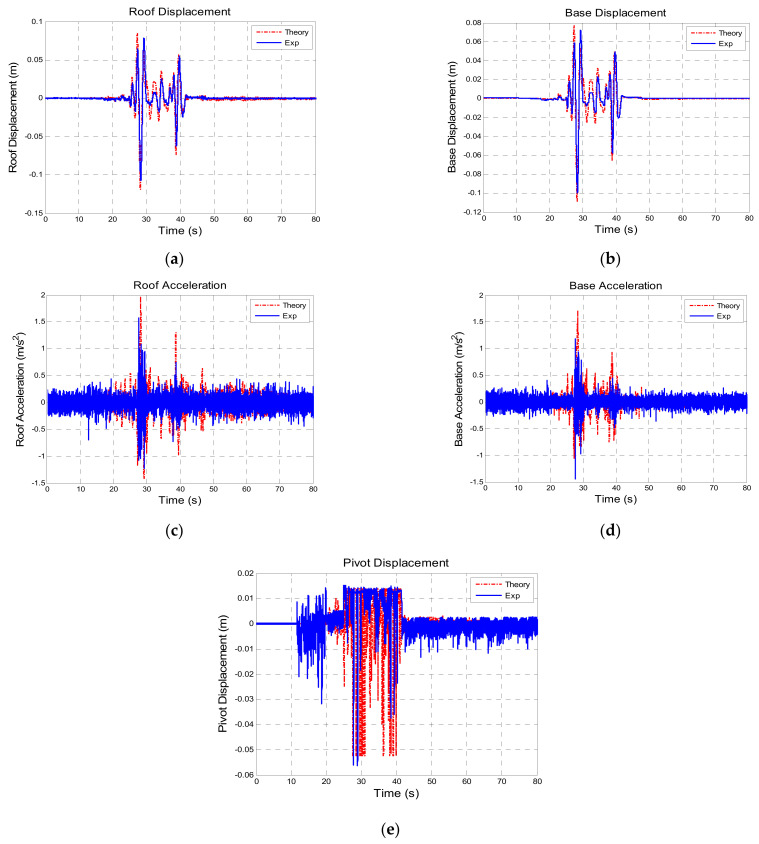
Comparison of the responses between theoretical and experimental values (TCU102-EW PGA = 0.1 g). (**a**) Superstructure displacement. (**b**) Isolation layer displacement. (**c**) Superstructure acceleration. (**d**) Isolation layer acceleration. (**e**) Pivot displacement.

To verify the accuracy and efficiency of proposed FPEEA control law, the experimental structural responses to Chi-Chi TCU102-EW (PGA = 0.10 g) under the FPEEA control were compared with the numerically obtained results. The experimental structural responses to Chi-Chi TCU102-EW (PGA = 0.10 g) under the FPEEA control were compared with the numerically obtained results. As displayed in Figure 7, the simulation and experiments yielded similar responses. Figure 7a,b displays the displacements of the superstructure and isolation layer, respectively. The maximum experimental displacements of the superstructure and isolation layer were 0.107 and 0.100 m, respectively. Furthermore, the displacements of the superstructure and isolation layer were obtained at 0.119 and 0.109 m, respectively, from the theoretical results.

The displacement response was smaller in the experiments than that in the simulations. The acceleration response from the experiment was close to the numerical results as shown in Figure 7c,d. It may be caused by the errors between the identified parameters of the numerical model and the practical situation. Meanwhile, the damping value of LSCIS may also be slightly underestimated. The maximum accelerations of the superstructure and isolation layer in the experiments were 1.573 and 1.440 m/s^2^, respectively, and the maximum simulated accelerations of the superstructure and isolation layer were 1.974 and 1.716 m/s^2^, respectively. The reduction of the acceleration of the isolated structure confirmed the efficiency of the FPEEA control in the experiments. As displayed in Figure 7e, the experimental and simulation results differed in terms of the pivot displacement of the isolation layer. The main reason for this phenomenon is that the depletion of the control over the pivot position during the experiments prevented the achievement of an expected displacement. Nevertheless, the structure was controlled efficiently, and the robustness of the system was demonstrated.

### 5.2. Comparison of Various Control Laws

**Figure 8:**
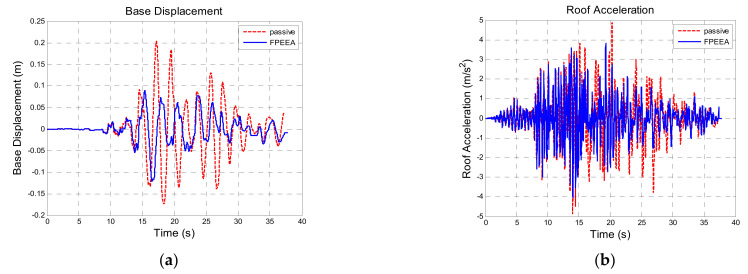
Comparison for the FPEEA and passive controls (Northridge, PGA = 0.3 g). (**a**) Isolation layer displacement. (**b**) Superstructure acceleration.

**Figure 9:**
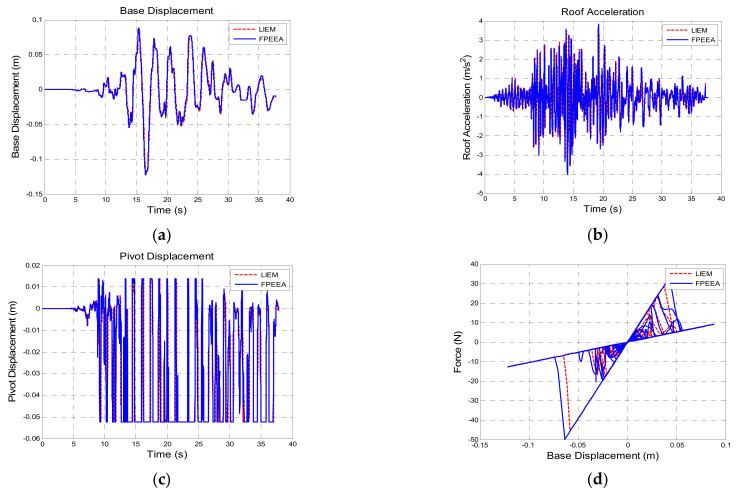
Comparison for the FPEEA and LIEM controls (Northridge, PGA = 0.3 g). (**a**) Isolation layer displacement. (**b**) Superstructure acceleration. (**c**) Pivot displacement. (**d**) Hysteresis loop.

**Figure 10:**
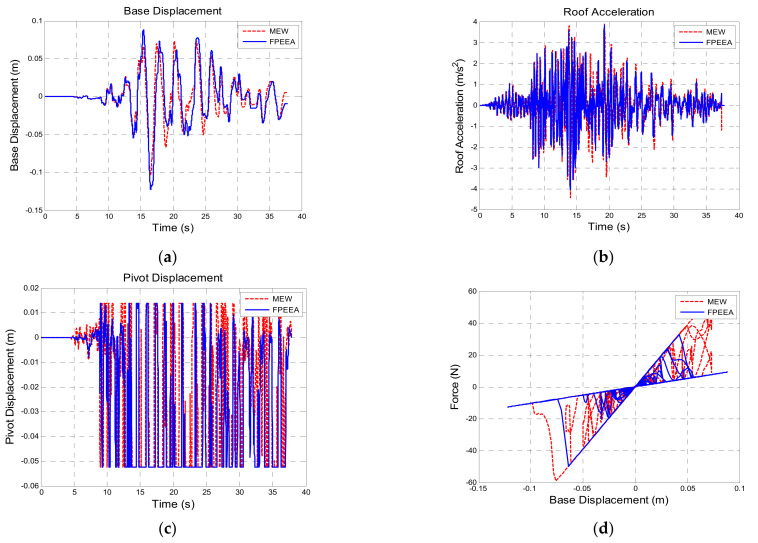
Comparison for the FPEEA and MEW controls (Northridge, PGA = 0.3 g). (**a**) Isolation layer displacement. (**b**) Superstructure acceleration. (**c**) Pivot displacement. (**d**) Hysteresis loop.

Table 10 shows a comparison of the structural responses of various algorithms to the Northridge earthquake (PGA = 0.3 g). As listed in the table, the simulated maximum displacement responses of the isolation layer under the passive control, the LIEM, the generic MEW method, and the FPEEA control were 0.203 m (100%), 0.121 m (59.7%), 0.103 m (50.7%), and 0.122 m (60.2%), respectively. The peak isolation layer displacement under the FPEEA control in the experiments was 0.117 m (57.6%). The FPEEA control and the MEW method reduced the displacement of the isolation layer in near-fault earthquakes by up to 60.2 and 57.6%, respectively. Moreover, the simulated peak acceleration responses for the passive control, the LIEM, the generic MEW method, and the FPEEA control were 4.87 m/s^2^ (100%), 3.967 m/s^2^ (81.5%), 4.042 m/s^2^ (83%), and 4.44 m/s^2^ (91.2%), respectively. The experimental peak acceleration of the superstructure under the FPEEA control was 4.562 m/s^2^ (93.7%). The FPEEA method outperformed the passive approach in terms of structural response as displayed in Figure 8. As depicted in the figure, the displacement of the isolation layer can be significantly suppressed by the FPEEA control than the passive control, and the superstructure acceleration can also be ameliorated effectively. Figure 9 indicates that the FPEEA control and the LIEM have comparable effects, and both reduce the isolation layer displacement and superstructure acceleration. The comparison of the structural responses to far-field earthquakes under the FPEEA control and the MEW method is further displayed in Figure 10. As depicted, the performance of the FPEEA method was on par with or superior to that of the MEW method. As the force required to control the response of the superstructure acceleration by the FPEEA control is relatively lower than the MEW control, the proposed control law is superior in controlling the structure response than the other listed control methods.

**Figure 11:**
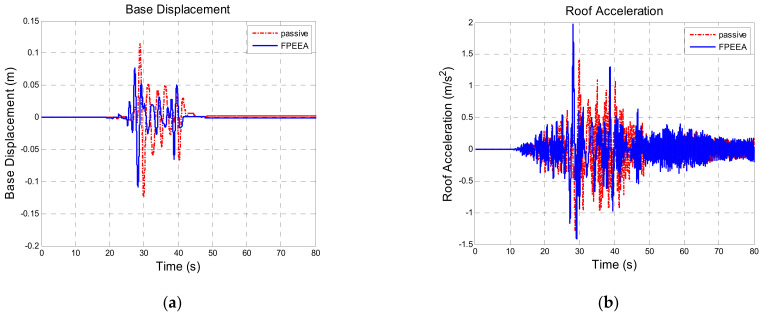
Comparison for the FPEEA and passive controls (TCU102, PGA = 0.1 g). (**a**) Isolation layer displacement. (**b**) Superstructure acceleration.

**Figure 12:**
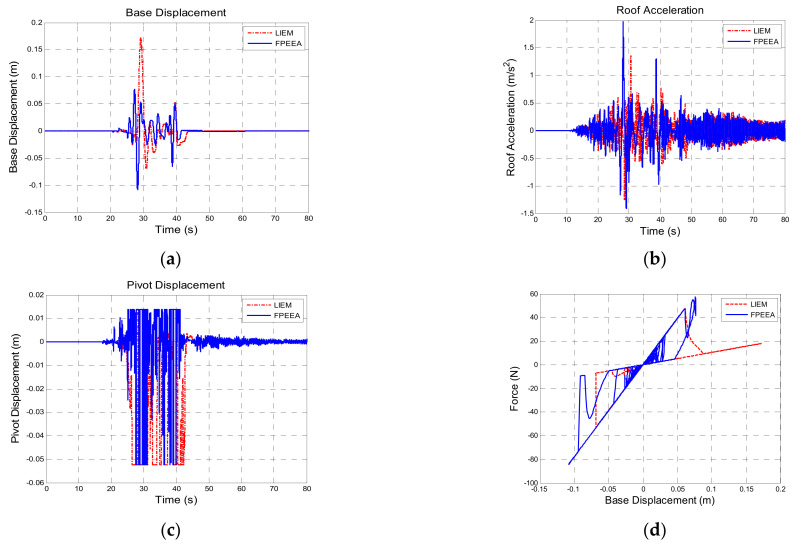
Comparison for the FPEEA and LIEM controls (TCU102, PGA = 0.1 g). (**a**) Isolation layer displacement. (**b**) Superstructure acceleration. (**c**) Pivot displacement. (**d**) Hysteresis loop.

**Figure 13:**
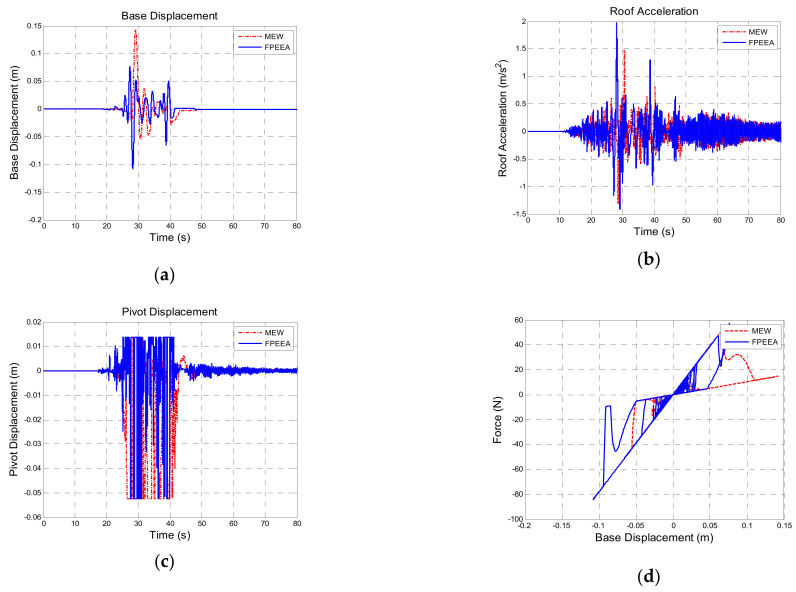
Comparison for the FPEEA and MEW controls (TCU102, PGA = 0.1 g). (**a**) Isolation layer displacement. (**b**) Superstructure acceleration. (**c**) Pivot displacement. (**d**) Hysteresis loop.

Table 11 presents a comparison of the structural responses to Chi-Chi TCU102-EW (PGA: 0.10 g). The theoretical peak displacements of the isolation layer under the passive control, the LIEM, the generic MEW method, and the FPEEA control were 0.124 m (100%), 0.172 m (138.7%), 0.142 m (114.1%), and 0.109 m (87.7%), respectively. However, the peak displacement of the isolation layer under the FPEEA control in the experiments was 0.100 m (80.5%). As indicated in Figure 11, the FPEEA control experimentally reduced the displacement of the isolation layer most. Figure 12 suggests that the FPEEA control outperformed the LIEM in terms of the isolation displacement, superstructure acceleration, pivot displacement, and the hysteresis loop effect. Figure 13 displays the comparison of the structural responses to near-fault earthquakes under the FPEEA control and the MEW method. This indicates that the isolation layer displacement under the near-fault earthquake can be alleviated effectively by the FPEEA control.

## 6. Conclusions

A FPEEA algorithm is proposed in this paper to reduce the structural responses to earthquakes, namely the isolation layer displacement and superstructure acceleration. The FPEEA control allows for the rapid determination of earthquake ground motion characteristics, after which potential energy weighting between near-fault and far-field earthquakes can be optimized and proportionally allocated. The performance of the proposed control system was verified against those of various isolated systems.

1. Simulation analysis revealed that the FPEEA control can reduce the displacement of the isolation layer under near-fault earthquakes. The isolation layer displacements under near-fault and far-field earthquakes were reduced by 80% and 60%, respectively.
2. A detailed comparison indicated that the FPEEA control outperformed the passive approach, LIEM, and generic MEW method in that it achieved greater acceleration response reduction.
3. Although the control effects of the FPEEA were comparable to those of the MEW method, the superstructure acceleration response was considerably lower under the FPEEA control than with the MEW method.
4. The experimental verification of the FPEEA control through the shake table tests conducted with the LSCIS indicated moderately higher reductions in the displacement and acceleration responses by the FPEEA than by the MEW.
5. In summary, the FPEEA control was effective in reducing the displacement response to near-fault earthquakes and in providing excellent structural control under far-field earthquakes.
6. Robustness of the proposed FPEEA control can be improved by increasing the ground motion database data in terms of near-fault and far-field earthquakes.

## Figures and Tables

**Table 1:** Energy distribution of near-fault earthquakes.

Earthquake and Station Name/Hz	0~1	1~2	2~3	3~4	4~5	Total
Chi-Chi, TCU078W	95.60%	1.40%	0.48%	0.76%	0.73%	98.97%
El Centro, H-180	38.80%	47.00%	7.54%	3.55%	1.91%	98.80%
Erzican, ERZ-NS	82.63%	11.88%	3.07%	1.52%	0.44%	99.54%
Imperial Valley, H-E06230	90.59%	5.93%	1.05%	0.73%	0.21%	98.52%
Kobe, Takatori-000	54.63%	30.25%	7.18%	2.87%	1.25%	96.19%
Kocaeli, YPT060	98.67%	0.71%	0.25%	0.13%	0.08%	99.84%
Loma Prieta, WVC270	96.31%	0.75%	0.36%	0.44%	0.21%	98.06%
Loma Prieta, LGP000	95.40%	2.73%	0.78%	0.34%	0.26%	99.51%
Northridge, RRS228	76.66%	22.30%	0.53%	0.19%	0.21%	99.90%
Parkfield, C02065	64.12%	17.51%	5.96%	3.61%	4.92%	96.12%
N. Palm Springs, NPS210	60.62%	23.13%	5.92%	5.51%	1.25%	96.44%
N. Palm Springs, WWT180	47.69%	18.59%	22.67%	3.54%	2.92%	95.41%
Morgan Hill, Halls Valley, HVR240	23.72%	56.49%	15.67%	1.64%	0.31%	97.82%
Morgan Hill, AND340	59.89%	18.21%	11.15%	4.44%	4.38%	98.06%
Loma Prieta, GIL337	66.62%	9.10%	14.28%	5.57%	1.80%	97.36%
Loma Prieta, G01000	85.70%	6.20%	3.22%	2.07%	0.41%	97.61%

**Table 2:** Energy distribution of far-field earthquakes.

Earthquake and Station Name/Hz	0~1	1~2	2~3	3~4	4~5	Total
Chalfant Valley, A-CVK000	63.12%	22.13%	5.24%	2.98%	1.64%	95.11%
Coalinga-01, H-C02000	73.83%	3.47%	4.26%	11.46%	1.99%	95.01%
Kern County, TAF111	94.63%	3.67%	0.46%	0.26%	0.57%	99.59%
Loma Prieta, FMS090	73.33%	2.71%	5.66%	8.83%	3.97%	94.50%
Loma Prieta, HSP090	56.92%	24.61%	7.59%	2.67%	2.32%	94.11%
Morgan Hill, SJB213	82.68%	7.34%	6.19%	0.79%	1.60%	98.61%
N.Palm Springs, Hesperia HES002	87.01%	3.38%	1.43%	1.83%	0.66%	94.32%
San Fernando, ORR291	37.87%	45.05%	13.54%	0.89%	0.95%	98.29%
Coalinga-01 Parkfield H-PG6000	7.72%	76.75%	7.04%	1.64%	2.22%	95.37%
N. Palm Springs H06360	43.65%	13.85%	28.61%	8.50%	1.20%	95.82%
Loma Prieta Hayward HWB310	93.55%	4.25%	0.66%	0.27%	0.27%	98.99%
Landers Yermo Fire Station YER360	83.64%	6.60%	3.25%	3.22%	0.48%	97.20%
Whittier Narrows-01 A-KAG315	20.15%	13.14%	15.14%	5.42%	16.97%	70.82%
Northridge-01, WAI290	82.79%	5.04%	2.83%	1.45%	1.38%	93.51%
Northridge-01, BA000	93.26%	1.29%	0.36%	2.65%	0.21%	97.76%
Northridge-01, STN110	71.14%	11.46%	3.93%	3.46%	2.69%	92.68%

**Table 3:** Near-fault and far-field earthquake numbers of different energy ratios.

Near to Far-Field/Energy Ratio	≥99%	≥98%	≥97%	<97%	<96%	<95%
Near-fault earthquake (number)	4	9	12	4	1	0
Far-field earthquake (number)	1	4	6	10	10	6
Total	5	13	18	14	11	6

**Table 4:** Probability of potential energy weighting to velocity–energy ratio.

$S_{1}$	$C_{1}$	$C_{2}$
$S_{1}\geq 99\%$	$\;\;\;C_{1}=\frac{4}{5}=80\%$	$C_{2}=\frac{1}{5}=20\%$
$98\%\leq S_{1}<99\%$	$\;\;\;\;\;\;\;\;\;\;\;C_{1}=\frac{9}{13}=69.23\%$	$\;\;\;\;\;\;\;\;C_{2}=\frac{4}{13}=30.77\%$
$97\%\leq S_{1}<98\%$	$\;\;\;\;\;\;\;\;\;\;C_{1}=\frac{12}{18}=66.67\%$	$\;\;\;\;\;\;\;\;C_{2}=\frac{6}{18}=33.33\%$
$96\%\leq S_{1}<97\%$	$\;\;\;\;\;\;\;\;\;\;C_{1}=\frac{4}{14}=28.57\%$	$\;\;\;\;\;\;\;\;C_{2}=\frac{10}{14}=71.43\%$
$95\%\leq S_{1}<96\%$	$\;\;\;\;\;C_{1}=\frac{1}{11}=9.1\%$	$\;\;\;\;\;\;C_{2}=\frac{10}{11}=90.9\%$
$S_{1}<95\%$	$C_{1}=\frac{0}{6}=0\%$	$\;\;C_{2}=\frac{6}{6}=100\%$

**Table 5:** Identified parameters of the LSCIS system.

	Property	Value
Superstructure	Mass (*m_s_*)	18.66 kg
	Damping (*c_s_*)	9.1104 N-s/m
	Stiffness (*k_s_*)	2780 N/m
	Natural frequency	1.95 Hz
isolation layer	Mass (*m_b_*)	38.445 kg
	Friction coefficient (µ)	0.002
	Stiffness (*k_r_*_0_)	500 N/m
	Stiffness incremental range of isolation layer *k_r_*	(1.5 k_r0_, 0.2 k_r0_)

**Table 6:** Parameters of each control law.

Seismic Isolation System	R	Potential Energy Weighing
Passive	X	X
LIEM	$10^{-8}$	X
MEW	$10^{-8}$	30
FPEEA	$10^{-8}$	$Q=C_{1}\cdot QE_{pn}+C_{2}\cdot QE_{pf}$

Note: $QE_{pn}=5$, $QE_{pf}=250\;;$ *C*_1_ and *C*_2_ are the probabilities of near-fault and far-field earthquakes, respectively; X indicates no value.

**Table 7:** Maximum responses of Whittier narrows-01 earthquake (PGA = 0.2g).

Seismic Isolation System	Superstructure Displacement (m)	Isolation Layer Displacement (m)	Superstructure Acceleration (m/s^2^)	Acceleration of Isolation Layer (m/s^2^)
Passive	0.021	0.019	0.430	0.369
	(1.00)	(1.00)	(1.00)	(1.00)
LIEM	0.016	0.015	0.372	0.303
	(0.766)	(0.779)	(0.866)	(0.819)
MEW	0.019	0.017	0.360	0.413
	(0.906)	(0.864)	(0.836)	(1.118)
FPEEA	0.016	0.015	0.362	0.355
	(0.777)	(0.773)	(0.843)	(0.960)

Note: Numbers in parentheses represent the relative response ratio to the passive mode.

**Table 8:** Maximum responses of TCU068- EW earthquake (PGA = 0.2 g).

Seismic Isolation System	Superstructure Displacement (m)	Isolation Layer Displacement (m)	Superstructure Acceleration (m/s^2^)	Acceleration of Isolation Layer (m/s^2^)
Passive	0.259	0.242	2.710	2.248
	(1.00)	(1.00)	(1.00)	(1.00)
LIEM	0.295	0.294	3.213	2.835
	(1.142)	(1.218)	(1.185)	(1.261)
MEW	0.238	0.236	3.448	2.926
	(0.921)	(0.976)	(1.272)	(1.301)
FPEEA	0.172	0.156	2.657	2.529
	(0.665)	(0.648)	(0.980)	(1.125)

Note: Numbers in the parentheses represent the relative response ratio to the passive mode.

**Table 9:** Identified parameters of the superstructure and isolation layer.

	Item	Value
Superstructure	Mass (*m_s_*)	20.52 kg
	Damping (*c_s_*)	9.1104 N-s/m
	Stiffness (*k_s_*)	2800 N/m
	Natural frequency	1.9 Hz
Isolation layer	Mass (*m_b_*)	38.445 kg
	Friction coefficient (μ)	0.002
	Stiffness (*k_r_*_0_)	520 N/m

**Table 10:** Maximum responses of the Northridge (PGA = 0.3 g) earthquake.

Isolation System	Superstructure Displacement (m)	Displacement of Isolation Layer (m)	Superstructure Acceleration (m/s^2^)	Acceleration of Isolation Layer (m/s^2^)
Passive (Simulation)	0.225	0.203	4.870	4.330
	(1.00)	(1.00)	(1.00)	(1.00)
LIEM(R = 10^−8^) (Simulation)	0.126	0.121	3.967	3.836
	(0.559)	(0.597)	(0.815)	(0.886)
MEW (Simulation)	0.105	0.103	4.440	4.006
	(0.465)	(0.507)	(0.912)	(0.925)
FPEEA (Simulation)	0.126	0.122	4.042	3.788
	(0.560)	(0.602)	(0.830)	(0.875)
FPEEA (Experiment)	0.130	0.117	4.562	4.587
	(0.578)	(0.576)	(0.937)	(1.059)

Note: Numbers in parentheses represent the ratio between passive and controlled responses.

**Table 11:** Maximum responses of TCU102 (PGA = 0.1 g) earthquake.

Seismic Isolation System	Superstructure Displacement (m)	Isolation Layer Displacement (m)	Superstructure Acceleration (m/s^2^)	Acceleration of Isolation Layer (m/s^2^)
Passive	0.134	0.124	1.415	1.238
(Simulation)	(1.00)	(1.00)	(1.00)	(1.00)
LIEM(R = 10^−8^)	0.170	0.172	1.348	1.289
(Simulation)	(1.268)	(1.387)	(0.953)	(1.042)
MEW	0.146	0.142	1.470	0.920
(Simulation)	(1.088)	(1.141)	(1.039)	(0.743)
FPEEA	0.119	0.109	1.974	1.716
(Simulation)	(0.886)	(0.877)	(1.395)	(1.387)
FPEEA	0.107	0.100	1.573	1.440
(Experiment)	(0.800)	(0.805)	(1.112)	(1.164)

Note: Numbers in parentheses represent the ratio between passive and controlled responses.
